# Complete mitochondrial genome and phylogenetic analysis of the copper shark *Carcharhinus brachyurus* (Günther, 1870)

**DOI:** 10.1080/23802359.2021.1920863

**Published:** 2021-05-18

**Authors:** Sang Wha Kim, Seon Young Park, Hyemin Kwon, Sib Sankar Giri, Sang Guen Kim, Jeong Woo Kang, Jun Kwon, Sung Bin Lee, Won Joon Jung, JunMo Lee, Se Chang Park, Ji Hyung Kim

**Affiliations:** a College of Veterinary Medicine and Research Institute for Veterinary Science, Laboratory of Aquatic Biomedicine, Seoul National University, Seoul, Republic of Korea; b Infectious Disease Research Center, Korea Research Institute of Bioscience and Biotechnology, Daejeon, Republic of Korea; c College of Agriculture and Life Science, Division of Animal and Dairy Sciences, Chungnam National University, Daejeon, Republic of Korea; d Department of Oceanography, Kyungpook National University, Daegu, Republic of Korea

**Keywords:** Shark, mitogenome, multigene phylogeny, conservation

## Abstract

Copper shark (*Carcharhinus brachyurus* Günther, 1870) is one of the most widely distributed but least known species in the family Carcharhinidae. Herein, we report the first complete mitogenome of *C. brachyurus*. The overall structure of the 16,704 bp *C. brachyurus* mitogenome was similar to that of other *Carcharhinus* species and showed the highest average nucleotide identity (97.1%) with the spinner shark (*Carcharhinus brevipinna*). Multigene phylogeny using 13 protein-coding genes (PCGs) in the mitogenome resolved *C. brachyurus* clustered with other species within the genus; the overall tree topology was congruent with recent phylogenetic studies of this species. These results provide important information for conservation genetics and further evolutionary studies of sharks.

Copper shark (*Carcharhinus brachyurus* Günther, 1870), in the family Carcharhinidae, is distributed in the equatorial temperate region of the eastern Atlantic, northwestern and eastern Pacific, southern Africa, Australia, and New Zealand (Compagno et al. 2005; Kim et al. 2019). In the IUNC Red List of threatened species, *C. brachyurus* is classified as ‘vulnerable’ (Huveneers et al. 2020). Various traits, including slow growth and late maturity render them vulnerable to anthropogenic activities (Drew et al. 2017). In the mitogenome of *C. brachyurus*, only the mitochondrial control region has been partially sequenced and used for phylogeographic analysis (Benavides et al. 2011). In this study, we analyzed the complete mitogenome sequence of *C. brachyurus*.

The specimens of *C. brachyurus* used in this study were collected from the Moseulpo fish markets in Jeju, Korea, in October 2017, as previously described (Kim et al. 2019). Muscle tissue samples were collected from fresh carcasses of adult female fish caught from the sea area between Gapa Island and Mara Island (33.147N, 126.269E). The collected specimens were transported and deposited at the College of Veterinary Medicine and Research Institute for Veterinary Science, Seoul National University (https://vet.snu.ac.kr/en, Prof. Se Chang Park, parksec@snu.ac.kr) under voucher number SNU-MO-0005.

Genomic DNA was extracted using the DNeasy Blood & Tissue Kit (Qiagen Korea Ltd., Seoul, Korea) according to the manufacturer’s instructions and subjected to direct PCR-based sequencing. Fifteen primer pairs, designed from the mitogenome of a related species in the family Carcharhinidae, the spinner shark (*Carcharhinu brevipinna*, NC_027081.1), were used to amplify the *C. brachyurus* mitogenome. All PCR primers and amplification conditions are listed in Supplementary Table 1. The obtained partial sequences of *C. brachyurus* were assembled using Geneious R11.1.5 (Kearse et al. 2012), and the final complete mitogenome was annotated as previously described (Kim et al. 2017).

The complete *C. brachyurus* mitogenome (MT995631) was 16,704 bp long, with a 61.7% A + T content. It consisted of a typical set of 37 genes (2 rRNAs, 22 tRNAs, and 13 protein-coding genes [PCGs]), and the overall structure was similar to the mitogenomes of other sharks in Carcharhinidae available in MitoFish (http://mitofish.aori.u-tokyo.ac.jp/). A putative D-loop (1061 bp, 66.4% A + T content) was located between *tRNA^Pro^* and *tRNA^Phe^*, and the replication origin (35 bp) was located on the H-strand between *tRNA^Asn^* and *tRNA^Cys^* **(**Supplementary Table 2**)**. The two rRNAs were 955 bp (12S rRNA) and 1676 bp (16S rRNA) long and separated by *tRNA^Val^*. The size of the 22 tRNAs varied from 67 bp (*tRNA^Ser^* and *tRNA^Cys^*) to 75 bp (*tRNA^Leu^*), with a total length of 1550 bp. Except for ND6, all PCGs were encoded on the H-strand of the genome. The 12 PCGs had the typical ATG initiation codon, whereas GTG was the initiation codon in *COX1*. Four types of stop codons were detected: TAA (*ND1*, *COX1*, *ATP8*, *COX3*, *ND4L*, and *ND5*), AGG (*ND6*), and the incomplete stop codons TA- (*ATP6* and *CYTB*) and T– (*ND2*, *COX2*, *ND3*, and *ND4*) (Supplementary Table 2).

Orthologous average nucleotide identity (OrthoANI) values were analyzed with other related sharks in the family Carcharhinidae as previously described (Kim et al. 2017). The overall OrthoANI values between *C. brachyurus* and other sharks in the genus *Carcharhinus* were >93%, with the highest value (97.1%) obtained for spinner shark (*C. brevipinna*, KM244770); other genera in the family showed <92% identity (Supplementary Table 3). Thirty-three complete mitogenomes of other related species in Carcharhinidae were obtained from GenBank and used for multigene phylogenetic analysis. The concatenated sequences of the 13 PCGs were aligned as previously described (Kim et al. 2017), and the maximum likelihood tree was reconstructed using MEGAX version 10.0 (Kumar et al. 2018). In the resultant tree, copper shark was clustered with other *Carcharhinus* species (Figure 1). Our tree topology was congruent with previous phylogenetic analyses based on nuclear or mitochondrial genes, which clustered blue shark (*Prionace glauca*) and whitetip reef shark (*Triaenodon obesus*) with other sharks in the genus *Carcharhinus* (Dosay-Akbulut 2008; Li et al. 2016), indicating that taxonomic assignment of the two species warrants further reevaluation. The mitogenome and associated genomic data of *C. brachyurus* provide important insights into biodiversity and address phylogenetic relationships within the genus *Carcharhinus*.

**Figure 1.**
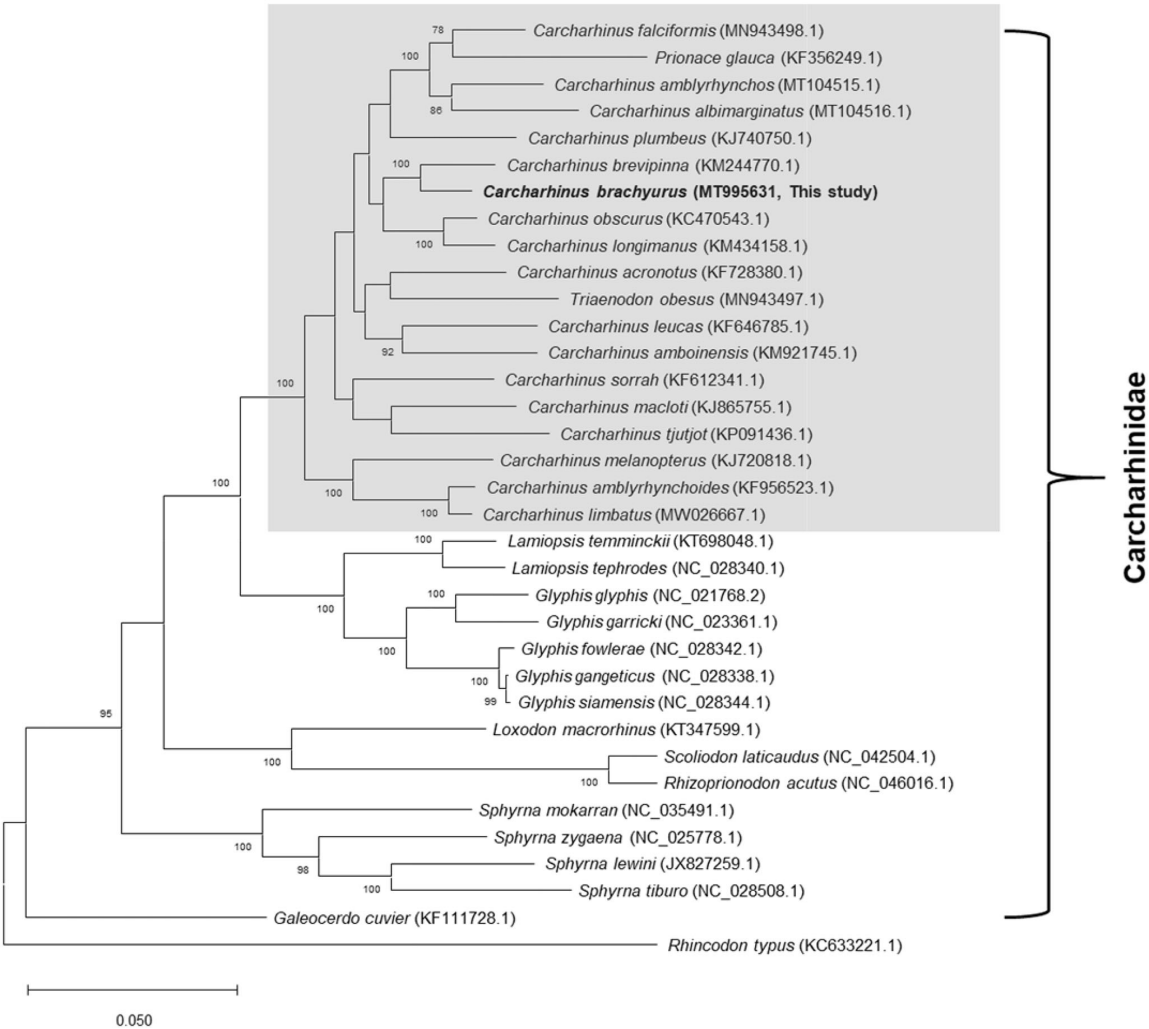
Maximum-likelihood phylogenetic tree based on the concatenated sequences of 13 protein-coding genes from the available Carcharhinidae mitogenomes. The gray box denotes the species currently placed in the genus *Carcharhinus* and other related taxa, including *Prionace glauca* and *Triaenodon obesus*. Mitochondrial genome of whale shark (*Rhincodon typus*, KC633221.1) was used as outgroup. Numbers at the branches indicate bootstrapping values obtained with 1,000 replicates, and only bootstrap values >70% are indicated. The scale bar represents 0.05 nucleotide substitutions per site.

## Supplementary Material

Supplemental Material

## Data Availability

The data that support the findings of this study are openly available in the NCBI GenBank database at https://www.ncbi.nlm.nih.gov, reference number MT995631.
