# Supplementary material for: Complete mitochondrial genome and phylogenetic analysis of the copper shark Carcharhinus brachyurus (Günther, 1870)
Source: Mitochondrial DNA B Resour. 2021 May 18;6(6):1659–61. doi: 10.1080/23802359.2021.1920863 (PMC8143640; doi:10.1080/23802359.2021.1920863)
Supplement: Supplemental Material [file TMDN_A_1920863_SM9932.zip › Suppl. Table 1. PCR Primers(Cb_final-312).docx]

**Supplementary Table 1.** PCR primers used for amplification of the copper shark (*Carcharhinus brachyurus*) mitogenome^*^.

| **No.** | **Primer name^**^** | **Sequence [5’-3’]** | **Tm (°C)** |
| --- | --- | --- | --- |
| 1 | K1_F | GGTCCTGGCCTTAGTGTTAAT | 55.9 |
|  | K1_R | CTTCGGAGTAGCTCGTTTAGTT | 56.3 |
| 2 | K2_F | CCCAGCGCAATAGCTTATGT | 55.4 |
|  | K2_R | CGAGGTCGTAAACCCTTTCTTC | 58.1 |
| 3 | K3_F | TCATCCACTTCCCAGGATATAAAC | 57.0 |
|  | K3_R | CGAATGGACCTCCTGCATATT | 55.9 |
| 4 | K4_F | GGTTCACCCTCCACACATTTA | 55.9 |
|  | K4_R | ATCCGAGGTTTGCGATTGAT | 53.4 |
| 5 | K5_F | CCCTCACTCAATCCCAACTTAT | 56.3 |
|  | K5_R | TCCGTGGGAAGGCTATATCT | 55.4 |
| 6 | K6_F | TTGGGCAACCTGGATCAC | 54.3 |
|  | K6_R | TGAGGCAAATGCTTCTCAAATG | 54.4 |
| 7 | K7_F | TTCCTAGGTCTCGCAGGTAT | 55.4 |
|  | K7_R | TGGTCAATTTCAGGGATTAGGT | 54.4 |
| 8 | K8_F | GACTCCCAACCACCCTTAAT | 55.4 |
|  | K8_R | GCTATGGTGGGCTCAAGTTA | 55.4 |
| 9 | K9_F | CCCTGAATTAGGCGGATGTT | 55.4 |
|  | K9_R | TGGATAGAGGTAGTGGTTCATAGT | 57.0 |
| 10 | K10_F | CACGGTTCCGACAAACTACA | 55.4 |
|  | K10_R | GAGAGGAGGTGATTAGGTGTTATTC | 58.9 |
| 11 | K11_F | CCTCCATCTCCAAACCTCATAG | 58.1 |
|  | K11_R | GAGGGTGAAGGCGAATTAAGA | 55.9 |
| 12 | K12_F | CCATGATTACCCTCAGCCATAG | 58.1 |
|  | K12_R | TGAACTGGGATGGAGGAAATG | 55.9 |
| 13 | K13_F | CGTAAAGCACCTCAAGACAAAC | 56.3 |
|  | K13_R | AAGAGAAGTATGGGTGGAATGG | 56.3 |
| 14 | K14_F | CTTCCACTTCCTACTCCCATTC | 58.1 |
|  | K14_R | AAACCAGGGCGGAAGATAAA | 53.4 |
| 15 | K15_F | CAAGAATGCCAGTCCTCTAGTT | 56.3 |
|  | K15_R | CGGCGGTATATAGACGGTAATG | 58.1 |
| 16 | K2-K3_intP | TGACCGTGCAAAGGTAGC | 54.3 |
| 17 | K3-K4_intP | TCAAGCCTTACCGTCTACAC | 55.4 |
| 18 | K4-K5_intP | ATTGGTCTAGCACCACTACAC | 55.9 |
| 19 | K5-K6_intP | TGCAATCCAACGTAAACAGC | 53.4 |
| 20 | K6-K7_intP | AGCCCACTTCCACTATGTTC | 55.4 |
| 21 | K7-K8_intP | CAGAAATTTGTGGTGCCAATC | 53.9 |
| 22 | K8-K9_intP | AATCCAAGCATATGTATTCGTCC | 54.9 |

^*^ PCR conditions for the amplification are as follows: a pre-denaturation at 95 ºC for 5 min, followed by amplification for 35 cycles at 95 ºC for 60 s, 56 ºC for 60 s and 72 ºC for 90 s, and a final extension at 72 ºC for 5 min.

^**^ PCR primers were designed according to the available mitochondrial genome of *Carcharhinus* *brevipinna* (NC_027081.1) in the GenBank database.
