# Supplementary material for: Complete mitochondrial genome and phylogenetic analysis of the copper shark Carcharhinus brachyurus (Günther, 1870)
Source: Mitochondrial DNA B Resour. 2021 May 18;6(6):1659–61. doi: 10.1080/23802359.2021.1920863 (PMC8143640; doi:10.1080/23802359.2021.1920863)
Supplement: Supplemental Material [file TMDN_A_1920863_SM9932.zip › Suppl. Table 3. genome ANI analysis(Cb_final-312).docx]

**Supplementary Table 3**. The complete mitogenomes of Carcharhinidae used for orthologous average nucleotide identity (OrthoANI) and phylogenetic analysis conducted in this study.

| **Family** | **Scientific name** | **Common name** | **GenBank No.** | **OrthoANI value (%)^*^** |
| --- | --- | --- | --- | --- |
| Carcharhinidae (n=34) | *Carcharhinus falciformis* | Silky shark | MN943498 | 95.1 |
|  | *Carcharhinus amblyrhynchos* | Grey reef shark | MT104515 | 94.7 |
|  | *Carcharhinus albimarginatus* | Silvertip shark | MT104516 | 94.1 |
|  | *Carcharhinus plumbeus* | Sandbar shark | KJ740750 | 95.1 |
|  | *Carcharhinus brevipinna* | Spinner shark | KM244770 | 97.1 |
|  | ***Carcharhinus brachyurus*** | **Copper shark** | **MT995631** | **This study** |
|  | *Carcharhinus obscurus* | Dusky shark | KC470543 | 96.1 |
|  | *Carcharhinus longimanus* | Oceanic whitetip shark | KM434158 | 95.8 |
|  | *Carcharhinus acronotus* | Blacknose shark | KF728380 | 95.2 |
|  | *Carcharhinus leucas* | Bull shark | KF646785 | 94.8 |
|  | *Carcharhinus amboinensis* | Pigeye shark | KM921745 | 94.6 |
|  | *Carcharhinus sorrah* | Spottail shark | KF612341 | 94.5 |
|  | *Carcharhinus macloti* | Hardnose shark | KJ865755 | 94.4 |
|  | *Carcharhinus tjutjot* | Whitecheek shark | KP091436 | 93.8 |
|  | *Carcharhinus melanopterus* | Blacktip reef shark | KJ720818 | 93.51 |
|  | *Carcharhinus amblyrhynchoides* | Graceful shark | KF956523 | 94.1 |
|  | *Carcharhinus limbatus* | Blacktip shark | MW026667 | 94.0 |
|  | *Prionace glauca* | Blue shark | KF356249 | 92.6 |
|  | *Triaenodon obesus* | Whitetip reef shark | MN943497 | 94.2 |
|  | *Lamiopsis temminckii* | Broadfin shark | KT698048 | 91.7 |
|  | *Lamiopsis tephrodes* | Borneo broadfin shark | NC_028340 | 91.6 |
|  | *Glyphis glyphis* | Speartooth shark | NC_021768 | 91.5 |
|  | *Glyphis garricki* | Northern river shark | NC_023361 | 91.3 |
|  | *Glyphis fowlerae* | Borneo river shark | NC_028342 | 91.5 |
|  | *Glyphis gangeticus* | Ganges shark | NC_028338 | 91.6 |
|  | *Glyphis siamensis* | Irrawaddy river shark | NC_028344 | 91.7 |
|  | *Loxodon macrorhinus* | Sliteye shark | KT347599 | 89.4 |
|  | *Scoliodon laticaudus* | Spadenose shark | NC_042504 | 86.7 |
|  | *Rhizoprionodon acutus* | Milk shark | NC_046016 | 86.7 |
|  | *Sphyrna mokarran* | Great hammerhead shark | NC_035491 | 88.1 |
|  | *Sphyrna zygaena* | Smooth hammerhead shark | NC_025778 | 88.2 |
|  | *Sphyrna lewini* | Scalloped hammerhead shark | JX827259 | 88.0 |
|  | *Sphyrna tiburo* | Bonnethead shark | NC_028508 | 87.8 |
|  | *Galeocerdo cuvier* | Tiger shark | KF111728 | 88.7 |

**^*^**The OrthoANI values were only determined between *C. brachyurus* (MT995631) and other related species in the Carcharhinus family.
