# Supplementary material for: Complete mitochondrial genome and phylogenetic analysis of the copper shark Carcharhinus brachyurus (Günther, 1870)
Source: Mitochondrial DNA B Resour. 2021 May 18;6(6):1659–61. doi: 10.1080/23802359.2021.1920863 (PMC8143640; doi:10.1080/23802359.2021.1920863)
Supplement: Supplemental Material [file TMDN_A_1920863_SM9932.zip › Suppl. Table 2. shark mitogenome(Cb_final-312).docx]

**Supplementary Table 2.** Mitochondrial genome characteristics of copper shark (*Carcharhinus brachyurus*).

| Gene | Position | | Size (bp) | Codon | | Intergenic nucleotides (bp) ^*^ | Coding strand^†^ |
| --- | --- | --- | --- | --- | --- | --- | --- |
|  | From | To |  | Start | Stop |  |  |
| *tRNA^Phe^* | 1 | 69 | 69 |  |  | 0 | H |
| *12S rRNA* | 70 | 1024 | 955 |  |  | 0 | H |
| *tRNA^Val^* | 1025 | 1096 | 72 |  |  | 0 | H |
| *16S rRNA* | 1097 | 2772 | 1676 |  |  | 0 | H |
| *tRNA^Leu(UUR)^* | 2773 | 2847 | 75 |  |  | 0 | H |
| *ND1* | 2848 | 3822 | 975 | ATG | TAA | 0 | H |
| *tRNA^Ile^* | 3823 | 3892 | 70 |  |  | 1 | H |
| *tRNA^Gln^* | 3894 | 3965 | 72 |  |  | -1 | L |
| *tRNA^Met^* | 3965 | 4033 | 69 |  |  | 0 | H |
| *ND2* | 4034 | 5078 | 1045 | ATG | T | 0 | H |
| *tRNA^Trp^* | 5079 | 5149 | 71 |  |  | 1 | H |
| *tRNA^Ala^* | 5151 | 5219 | 69 |  |  | 0 | L |
| *tRNA^Asn^* | 5220 | 5292 | 73 |  |  | 0 | L |
| Rep origin | 5293 | 5327 | 35 |  |  | 0 | H |
| *tRNA^Cys^* | 5328 | 5394 | 67 |  |  | 1 | L |
| *tRNA^Tyr^* | 5396 | 5464 | 69 |  |  | 1 | L |
| *COX1* | 5466 | 7022 | 1557 | GTG | TAA | 0 | H |
| *tRNA^Ser(UCN)^* | 7023 | 7093 | 71 |  |  | 3 | L |
| *tRNA^Asp^* | 7097 | 7166 | 70 |  |  | 7 | H |
| *COX2* | 7174 | 7864 | 691 | ATG | T | 0 | H |
| *tRNA^Lys^* | 7865 | 7938 | 74 |  |  | 1 | H |
| *ATP8* | 7940 | 8107 | 168 | ATG | TAA | -10 | H |
| *ATP6* | 8098 | 8780 | 683 | ATG | TA | 0 | H |
| *COX3* | 8781 | 9566 | 786 | ATG | TAA | 2 | H |
| *tRNA^Gly^* | 9569 | 9638 | 70 |  |  | 0 | H |
| *ND3* | 9639 | 9987 | 349 | ATG | T | 0 | H |
| *tRNA^Arg^* | 9988 | 10057 | 70 |  |  | 0 | H |
| *ND4L* | 10058 | 10354 | 297 | ATG | TAA | -7 | H |
| *ND4* | 10348 | 11728 | 1381 | ATG | T | 0 | H |
| *tRNA^His^* | 11729 | 11797 | 69 |  |  | 0 | H |
| *tRNA^Ser(AGY)^* | 11798 | 11864 | 67 |  |  | 0 | H |
| *tRNA^Leu(CUN)^* | 11865 | 11936 | 72 |  |  | 0 | H |
| *ND5* | 11937 | 13766 | 1830 | ATG | TAA | -5 | H |
| *ND6* | 13762 | 14283 | 522 | ATG | AGG | 0 | L |
| *tRNA^Glu^* | 14284 | 14353 | 70 |  |  | 2 | L |
| *CYTB* | 14356 | 15500 | 1145 | ATG | TA | 0 | H |
| *tRNA^Thr^* | 15501 | 15572 | 72 |  |  | 2 | H |
| *tRNA^Pro^* | 15575 | 15643 | 69 |  |  | 0 | L |
| D-loop | 15644 | 16704 | 1061 |  |  | 0 | - |

^*^ Numbers correspond to the nucleotides separating different genes. Negative numbers indicate overlapping nucleotides between adjacent genes.

^†^ H and L denote heavy and light strands, respectively.
